# Immune profiles of male giant panda (*Ailuropoda melanoleuca*) during the breeding season

**DOI:** 10.1186/s12864-021-07456-x

**Published:** 2021-02-28

**Authors:** Haibo Shen, Caiwu Li, Ming He, Yan Huang, Jing Wang, Minglei Wang, Bisong Yue, Xiuyue Zhang

**Affiliations:** 1 Key Laboratory of Bio-resources and Eco-environment, Ministry of Education, College of Life Science, Sichuan University, No. 24 South Section 1, Yihuan Road, Chengdu, 610065 Sichuan China; 2 Key Laboratory of State Forestry and Grassland Administration on Conservation Biology of Rare Animals in The Giant Panda National Park, China Conservation and Research Center for the Giant Panda, Dujiangyan, 611830 Sichuan PR China; 3 Sichuan Key Laboratory of Conservation Biology on Endangered Wildlife, College of Life Sciences, Sichuan University, Chengdu, 610064 PR China

**Keywords:** Male giant panda, Immune change, Breeding season, RNA-seq

## Abstract

**Background:**

The giant panda (*Ailuropoda melanoleuca*) is a threatened endemic Chinese species and a flagship species of national and global conservation concern. Life history theory proposes that reproduction and immunity can be mutually constraining and interrelated. Knowledge of immunity changes of male giant pandas during the breeding season is limited.

**Results:**

Here, we researched peripheral blood gene expression profiles associated with immunity. Thirteen captive giant pandas, ranging from 9 to 11 years old, were divided into two groups based on their reproductive status. We identified 318 up-regulated DEGs and 43 down-regulated DEGs, which were enriched in 87 GO terms and 6 KEGG pathways. Additionally, we obtained 45 immune-related genes with altered expression, mostly up-regulated, and identified four hub genes *HSPA4*, *SUGT1*, *SOD1*, and *IL1B* in PPI analysis. These 45 genes were related to pattern recognition receptors, autophagy, peroxisome, proteasome, natural killer cell, antigen processing and presentation. *SUGT1* and *IL1B* were related to pattern recognition receptors. *HSP90AA1* was the most up-regulated gene and is a member of heat shock protein 90 family. HSP90 contributes to the translocation of extracellular antigen. *KLRD1* encodes CD94, whose complex is an inhibitor of the cytotoxic activity of NK cells, was down-regulated. *IGIP,* which has the capability of inducing IgA production by B cells, was down-regulated, suggesting low concentration of IgA in male giant pandas. Our results suggest that most immune-related genes were up-regulated and more related to innate immune than adaptive immune.

**Conclusions:**

Our results indicated that breeding male giant pandas presented an immunoenhancement in innate immunity, enhanced antigen presentation and processing in cellular immunity compared to non-breeding males. The humoral immunity of male giant pandas may show a tendency to decrease during the breeding season. This study will provide a foundation for further studies of immunity and reproduction in male giant pandas.

**Supplementary Information:**

The online version contains supplementary material available at 10.1186/s12864-021-07456-x.

## Background

Reproductive activity, with a high metabolic cost, is associated with body and immunological conditions [1]. Life history theory proposes that reproduction and immunity can be mutually constraining and interrelated due to the optimal allocation of limited nutrients and energy [2]. An immune response is responsible for a substantial energetic costs [3]. Energy investment in reproduction leads to a corresponding decrease in immune investment, resulting in a trade-off between the two [4]. It is challenging to elucidate the underlying reproduction and immunity trade-off mechanisms, while it is easy to observe and record immune traits during reproduction [5]. Many male-focused studies on a variety of species have documented that males have reduced innate immunity and lower cellular immunity during reproductive periods [3, 6, 7]. However, several studies have found that during reproduction there is immunoenhancement of cellular immunity and higher resistance against bacteria [8, 9].

The giant panda (*Ailuropoda melanoleuca*), known as China’s national treasure, is a Chinese flagship species, a threatened species of global concern and a worldwide symbol of conservation [10]. Our research group previously showed that maternal giant panda immunity undergoes dramatic changes during estrus, early pregnancy, and late pregnancy [11]. Males also undergo reproductive changes, although not as dramatic as females. Male giant pandas reach sexual maturity at approximately 8 years old and undergo increases in testes volume, androgen concentrations and sperm production each breeding season thereafter [12]. According to previous studies, male giant pandas’ reproductive cycle was divided into three periods: breeding season (February–May), prebreeding season (October–January) and non-breeding season (June–September) [13]. However, most studies focused on the reproductive behaviors [13–15] and little is known about the immune change of male giant panda during the breeding season. The greater understanding of giant panda maternal immunity has allowed better health care and disease prevention and therefore there is a real need for male reproductive immune system research.

Peripheral blood is the important vehicle of the immune system. Many methods can quantify and measure immune traits from blood samples, such as total leukocyte counts [16], cytokines [17], complement and lysozyme activity [18] and phagocytosis activity [19]. Alternatively, immunocompetence can be quantified by phytohaemagglutinin challenge test [8] and sheep red blood cells injection test [20]. Measuring only one or few immune parameters, which treats the immune system like a “black box” [4], may be insufficient to reflect the overall immune system status and change. Transcriptome analysis is a robust tool that investigates the immune system by assessing transcript abundance changes in blood on a genome-wide scale, earning its place in immune function study [21]. Therefore, we aimed to compare the immune profiles of breeding and non-breeding male giant pandas. Transcriptome analysis was used to quantitatively evaluate transcript levels and identify the immune-related differentially expressed genes (DEGs) and pathways. This study will provide a foundation for further reproductive immunity and disease prevention studies on breeding male giant pandas.

## Results

### Reads sequencing and processing

Raw Illumina RNA-seq data were converted into clean reads data. All raw data has been deposited at NCBI Sequence Read Archive under the project accession no. PRJNA631846. A total of 95.49 Gb of paired-end clean data were generated. FastQC showed that the percent of Q30 was above 85%.

**Fig. 1:**
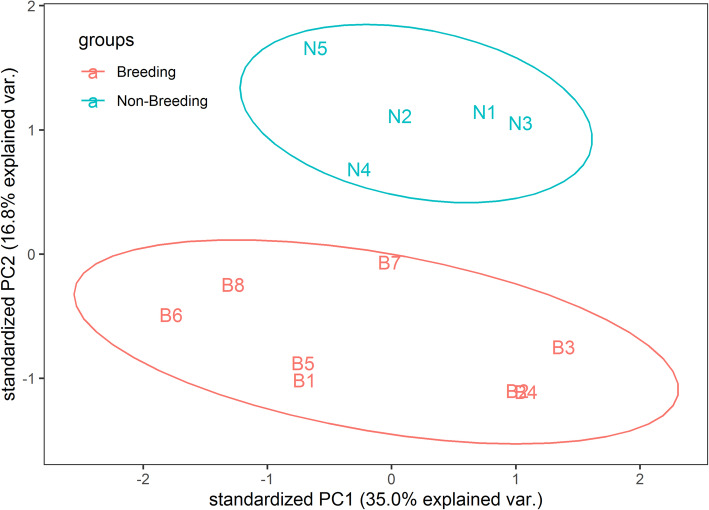
PCA analysis of 13 samples

HISAT2 mapping results revealed all sample overall alignment rates were between 86 and 90%. Read summarization counted by program featureCounts was converted into a numerical matrix. PCA results based on normalized matrix demonstrated that the 13 samples were divided into two groups from different dimensions (Fig. 1). Giant pandas in the breeding season were clustered into one group, while the non-breeding individuals were clustered into another group.

### Identification of DEGs

We detected 1128 genes with changed expression level, given the FDR threshold (Additional file 1: Table S1). By setting a cutoff of log_2_FC, 318 up-regulated DEGs and 43 down-regulated DEGs were identified in the breeding season compared to the non-breeding season. Two hundred seventy-five in 318 upregulated genes had annotations, while 33 in 43 down-regulated genes had annotations.

In the top 10 up-regulated DEGs, eight genes were involved in genetic information processing, mainly in transcription. *HSP90AA1* is a member of heat shock protein 90 family. *HSP90AA1* participates in numerous immune processes, such as antigen processing and presentation, Th17 cell differentiation, and NOD-like receptor signaling pathway. *PSMD7* encodes proteasome 26S subunit. Proteasome plays a great role in innate and adaptive immune responses.

In top 10 down-regulated DEGs, five genes were related to genetic information processing, such as transcription, translation, and protein export. *IGIP* (Immunoglobulin A inducing protein) belongs to the Immunoglobulin A regulatory factors family. *KLRD1* (killer cell lectin-like receptor subfamily D member 1) is associated with natural killer cell immunity.

### Gene ontology enrichment of DEGs

**Fig. 2:**
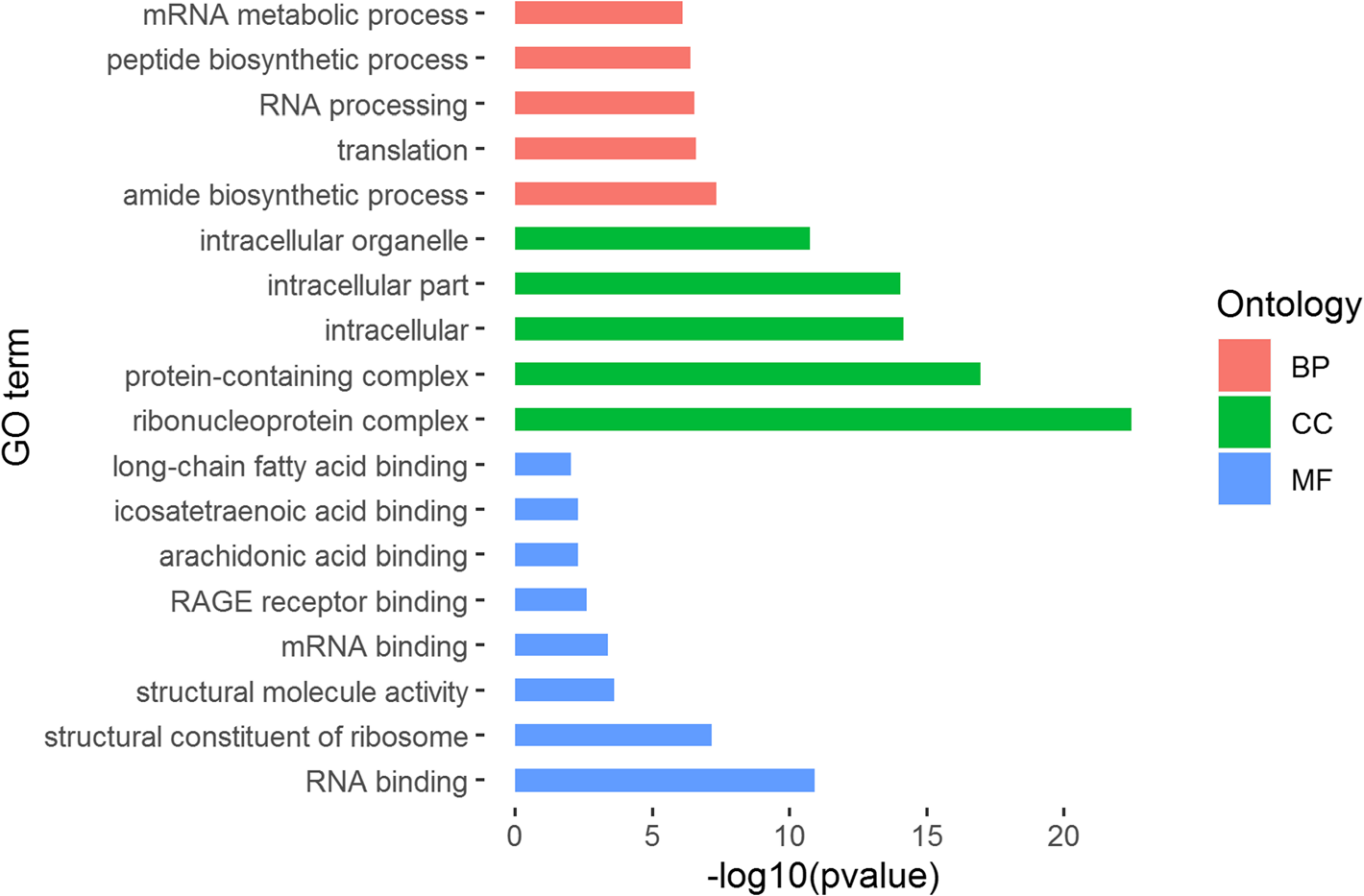
Partial GO enrichment of up-regulated DEGs

**Fig. 3:**
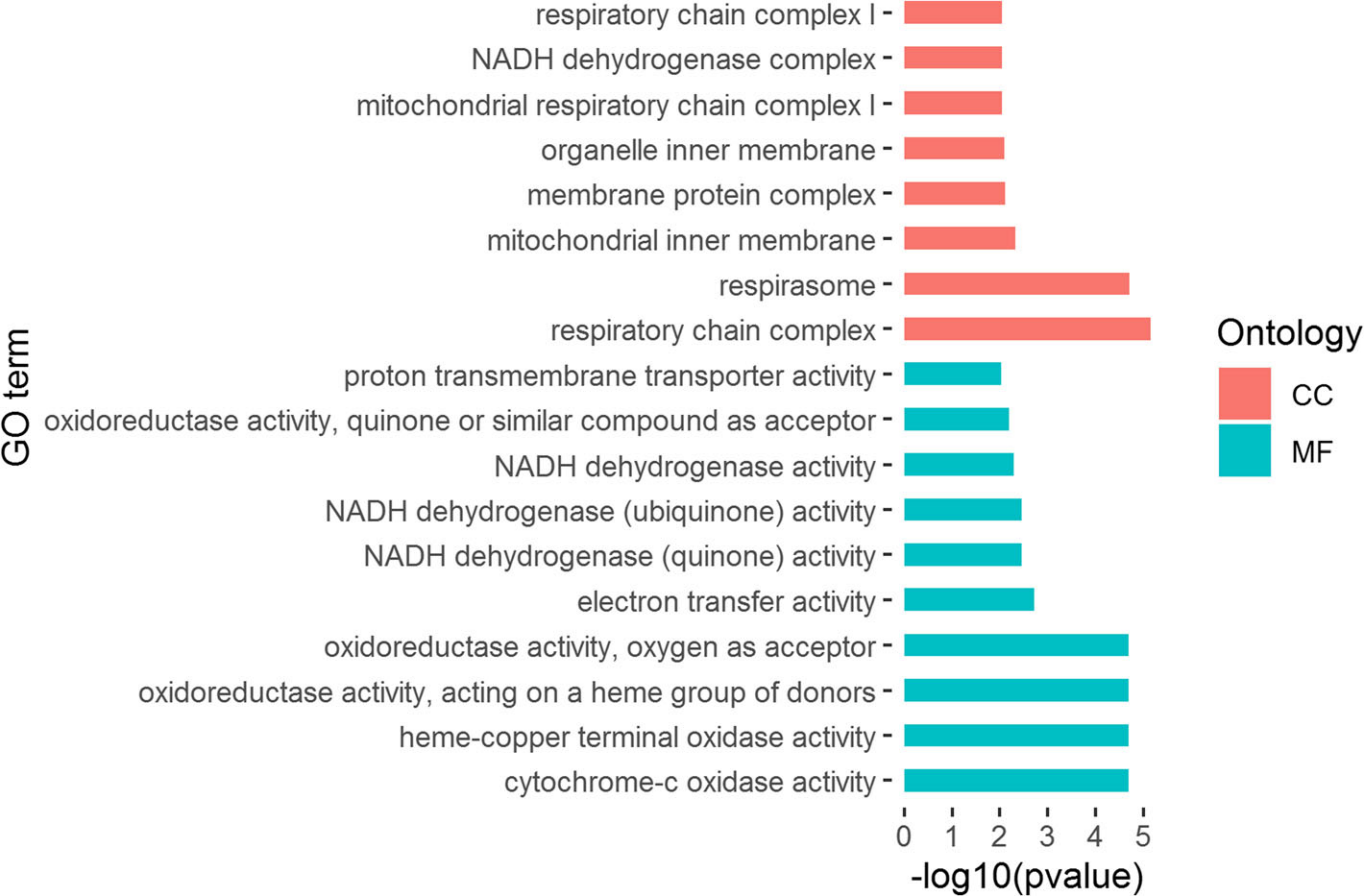
GO enrichment of down-regulated DEGs

Up-regulated DEGs were enriched in 69 GO terms, being 22 terms in biological process, 39 terms in cellular component and 8 terms in molecular function (Fig. 2). Down-regulated DEGs were enriched in 18 GO terms, which were 8 terms in cellular component and 10 terms in molecular function (Fig. 3). All GO term enrichments are shown in Additional file 2: Table S2. There were some overlap top-level cellular component terms between up-regulated DEGs and down-regulated DEGs, such as protein-containing complex (GO:0032991), cell (GO:0005623), cell part (GO:0044464) and organelle (GO:0043226). For down-regulated DEGs, the most significantly enriched molecular function term was cytochrome-c oxidase activity (GO:0004129). For up-regulated DEGs, the enriched GO terms in molecular function included gene expression (GO:0010467) and RAGE receptor binding (GO:0050786) which was associate with immune and inflammatory responses.

### KEGG pathway enrichment of DEGs

**Fig. 4:**
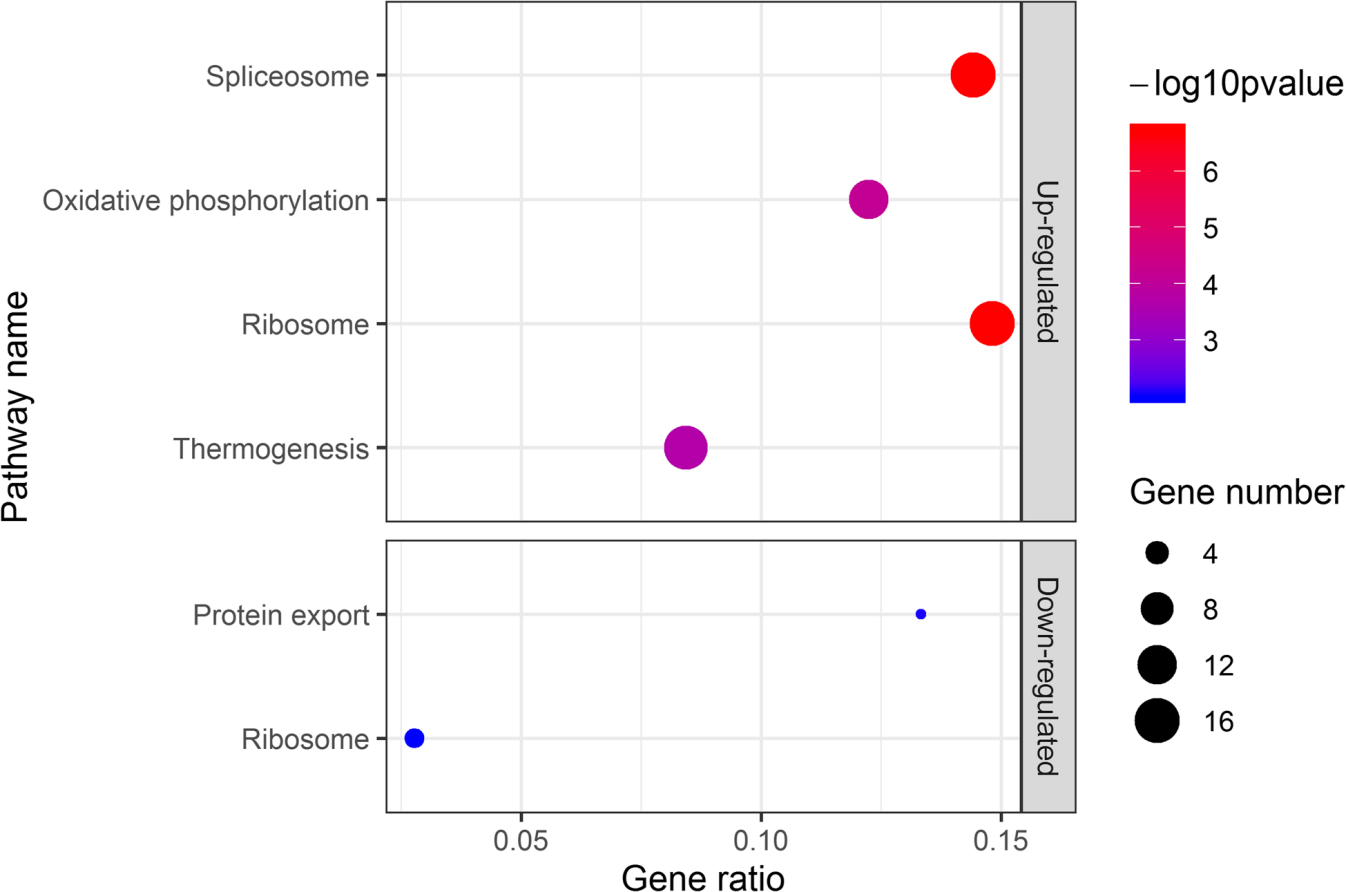
KEGG enrichment of up-regulated and down-regulated DEGs

Using an overrepresented analysis, we performed KEGG enrichment analysis for further understanding of DEGs. Up-regulated DEGs and down-regulated DEGs were enriched in four and two KEGG pathways respectively (Fig. 4). Up-regulated genes were enriched in ribosome (aml03010), spliceosome (aml03040), oxidative phosphorylation (aml00190) and thermogenesis (aml04714) pathways. Ribosome and spliceosome pathways were associated with genetic information processing. Thermogenesis was the child term of environmental adaptation pathway. Oxidative phosphorylation was the downstream term of thermogenesis. When focusing on down-regulated genes, we found the protein export (aml03060) and ribosome (aml03010) pathway were significantly enriched. Protein export was the child term of genetic information processing pathway.

**Fig. 5:**
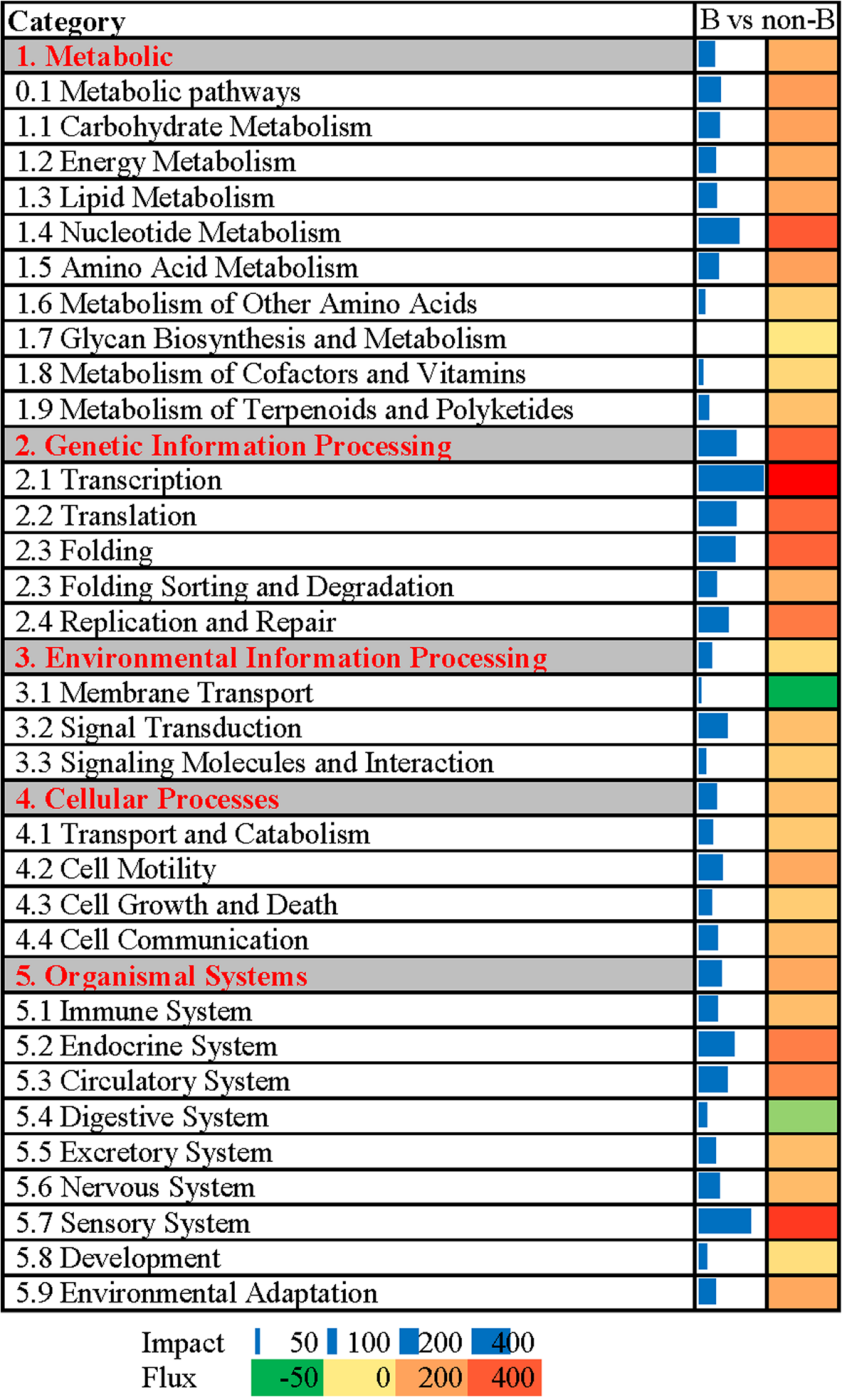
Summary of the main categories and subcategories of KEGG pathways analyzed by DIA. On the right are the bar denoting the overall impact (in blue) and the shade denoting the effect on the pathway (from green (inhibited)—to red (activated)). Darker the color larger the activation (if red) or inhibition (if green) of the pathway. “B” represents the “breeding season”. “non-B” represents the “non-breeding season”

**Fig. 6:**
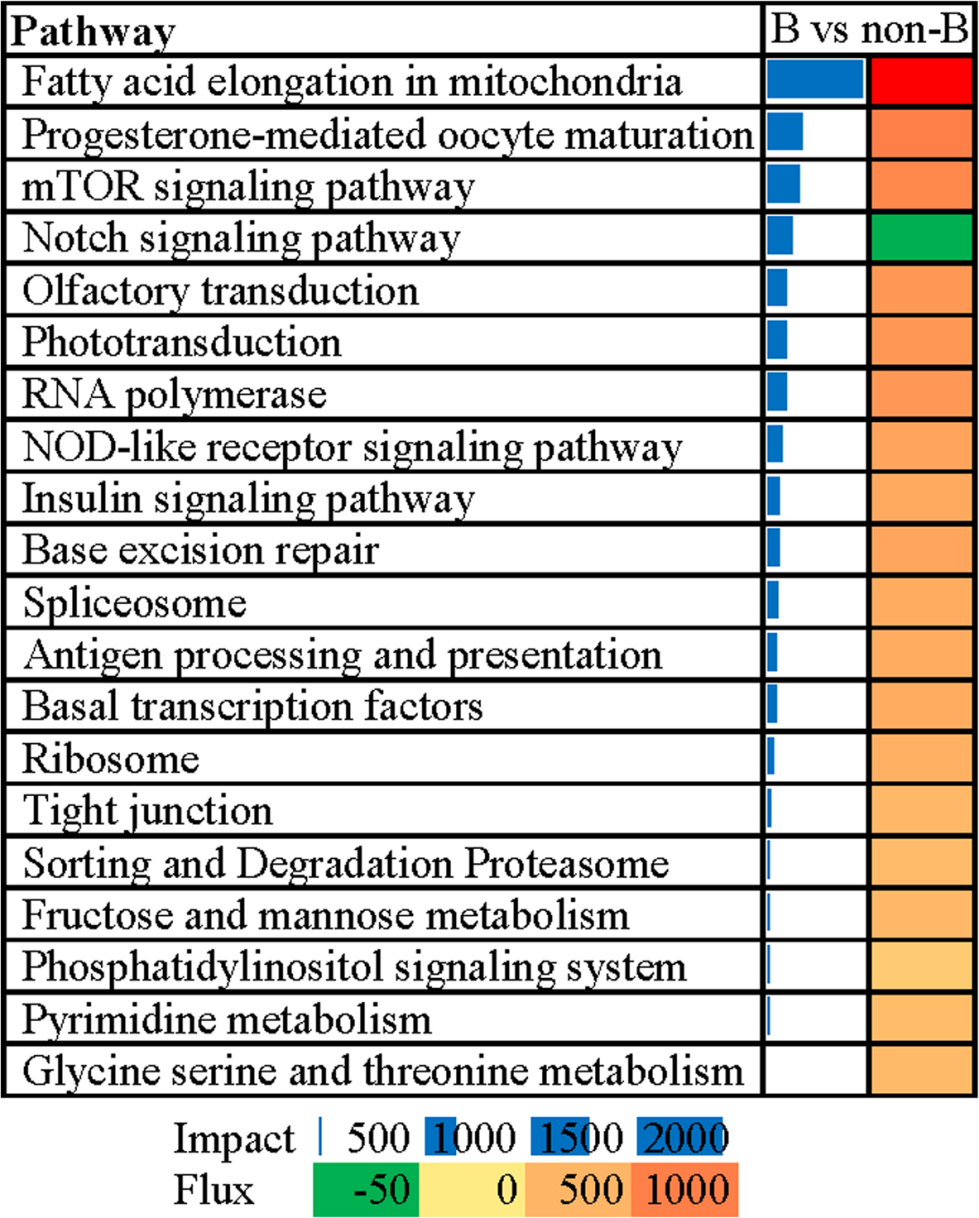
The 20 most impacted pathways analyzed by DIA. On the right are the bar denoting the overall impact (in blue) and the shade denoting the effect on the pathway (from green (inhibited)—to red (activated)). Darker the color larger the activation (if red) or inhibition (if green) of the pathway. “B” represents the “breeding season”. “non-B” represents the “non-breeding season”

We identified the biological impact of the breeding stage and the direction of the impact using a Dynamic Impact Approach (DIA). The summary of KEGG main categories and sub-categories is shown in Fig. 5. Among the main categories of KEGG, the category “Genetic Information Processing” was the most impacted, followed by “Organismal Systems” and “Cellular Processes”. Except for inhibition of “Membrane Transport” and “Digestive System”, the flux values of sub-categories were activated. The sub-category “Transcription” was the most impacted, followed by “Sensory System”. The top 20 most-impacted pathways are shown in Fig. 6. The most impacted pathway was “Fatty acid elongation in mitochondria” followed by “Progesterone-mediated oocyte maturation”. “Notch signaling pathway” was the only inhibited pathway. Among the top 20 pathways, “NOD-like receptor signaling pathway” and “Antigen processing and presentation” were associated with the immune system.

### Expression of immune-associated genes

**Fig. 7:**
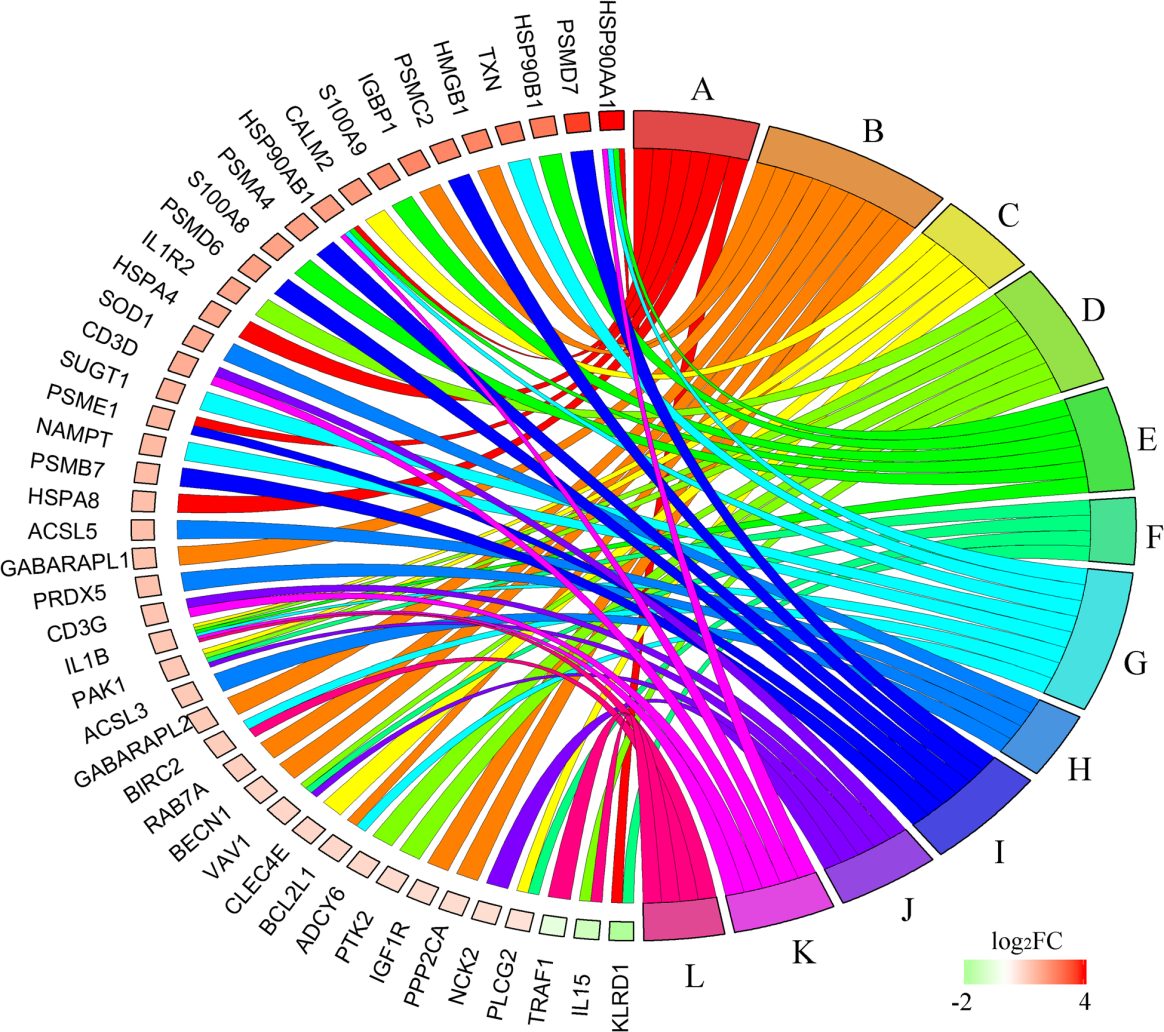
Chord diagram of categories of immune-associated genes. Genes were divided into twelve branches according to KEGG pathways: A –Antigen processing and presentation; B –Autophagy; C –C-type lectin receptor signaling pathway; D –Cytokine and Chemokine; E –IL-17 signaling pathway; F –Natural killer cell mediated cytotoxicity; G –NOD-like receptor signaling pathway; H –Peroxisome; I –Proteasome; J –T cell receptor signaling pathway; K –Th17 cell differentiation; L –TNF signaling pathway

**Fig. 8:**
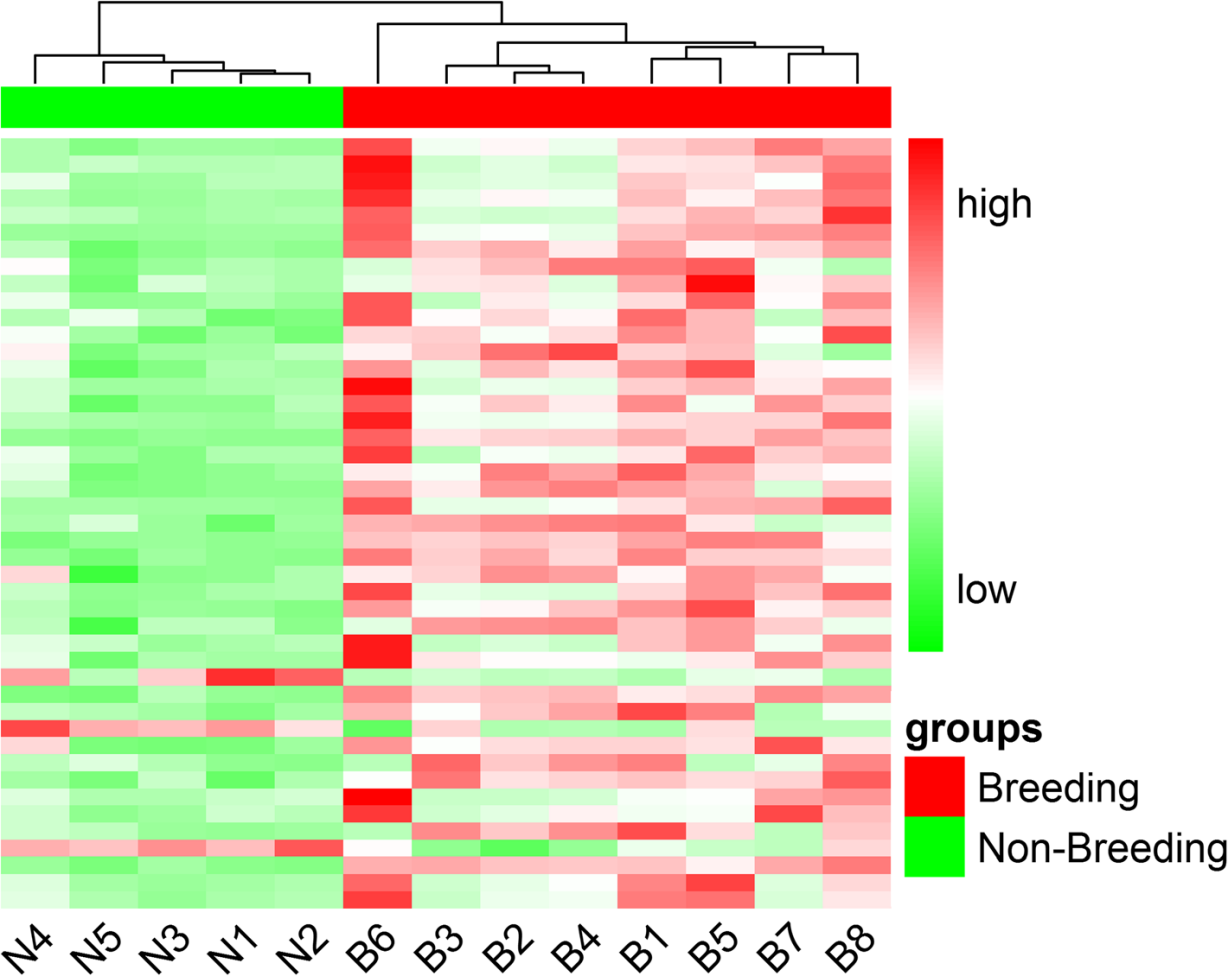
Heat map plot of 45 immune-related genes. The expression values of 13 pandas are presented after being centered and scaled in the row direction. Each column represents a specimen and each row represents a gene. Red color indicates genes which were up-regulated and green color indicates genes which were down-regulated

We obtained 45 immune-related genes and clustered them into 12 key categories according to KEGG annotation (Fig. 7). We also plotted the heatmap of immune-related genes to visualize their expression in all samples (Fig. 8). These categories were roughly divided into innate immune entries and adaptive immune entries. Innate immune system entries consisted of C-type lectin receptor, NOD-like receptor, autophagy, peroxisome, proteasome, natural killer cell, cytokine and chemokine, and TNF signaling pathway. Adaptive immune entries consisted of antigen processing and presentation, T cell receptor signaling pathway, Th17 cell differentiation, and IL-17 signaling pathway.

The expression trends of 45 genes were consistent, mostly up-regulated, while *KLRD1* (killer cell lectin-like receptor subfamily D member 1), *IL15* (interleukin 15) and *TRAF1* (TNF receptor-associated factor 1) were down-regulated. *CLEC4E* (C-type lectin domain family 4 member E) is a member of the C-type lectin receptor signaling pathway. *NAMPT* (nicotinamide phosphoribosyltransferase) and *GABARAPL1* (GABA type A receptor associated protein like 1) participate in the NOD-like receptor signaling pathway. *BECN1* (Beclin1), *PRDX5* (peroxiredoxin 5) and *PSME1* (PA28 alpha) shows the great function in autophagy, peroxisome and proteasome respectively. *VAV1* (guanine nucleotide exchange factor) and *PLCG2* (phosphatidylinositol phospholipase C gamma-2) are linked to natural killer cell. *IL15* together with *IL1R*2 (interleukin 1 receptor type 2) are two important cytokines. Lastly, *CD3D* (T-cell surface glycoprotein CD3 delta chain) and *CD3G* (T-cell surface glycoprotein CD3 gamma chain) are associated with T cell receptor signaling pathway and Th17 cell differentiation.

### Protein-protein interaction network of immune-associated genes

**Fig. 9:**
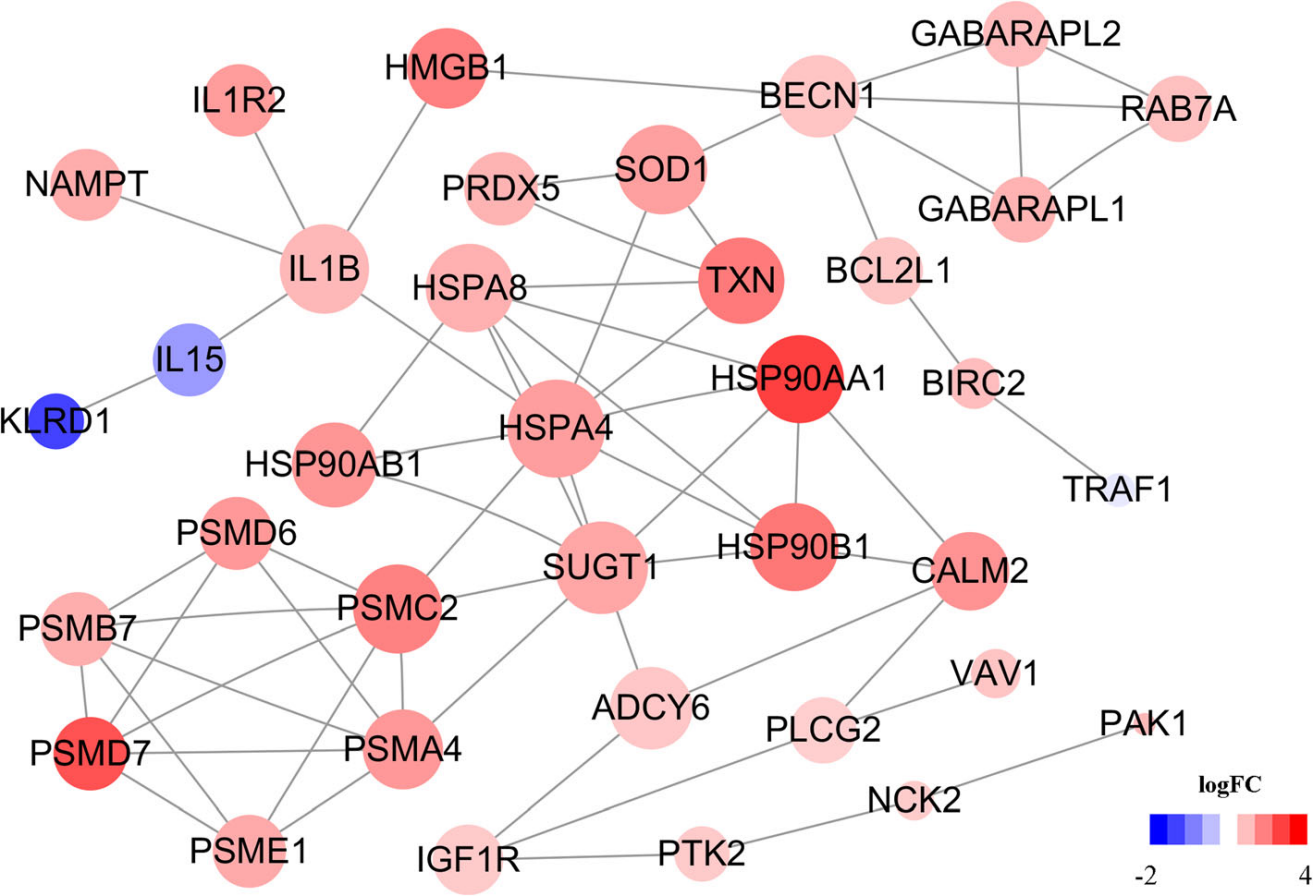
Protein-protein interaction network of DEGs. The nodes represent proteins and edges represent pair-wise interactions. The size of the nodes is score of the protein calculated by cytoHubba. The red nodes represent up-regulated proteins. The blue nodes represent down-regulated proteins

All immune-associated genes were converted into proteins by STRING. A total of 64 interaction edges between 36 nodes were extracted from the database after removing 9 isolated nodes. What’s more, we calculated the hub genes by using cytoHubba. The score of 36 genes were calculated by topological analysis methods. (Additional file 3: Table S3). We plotted the network diagram to illustrate interaction among proteins (Fig. 9). *HSPA4* (heat shock 70 kDa protein 4), *SUGT1* (*SGT1* homolog), *SOD1* (superoxide dismutase 1), and *IL1B* (interleukin 1 beta) were at important position within the interaction network.

### Real-time quantitative PCR (qRT-PCR) validation

**Fig. 10:**
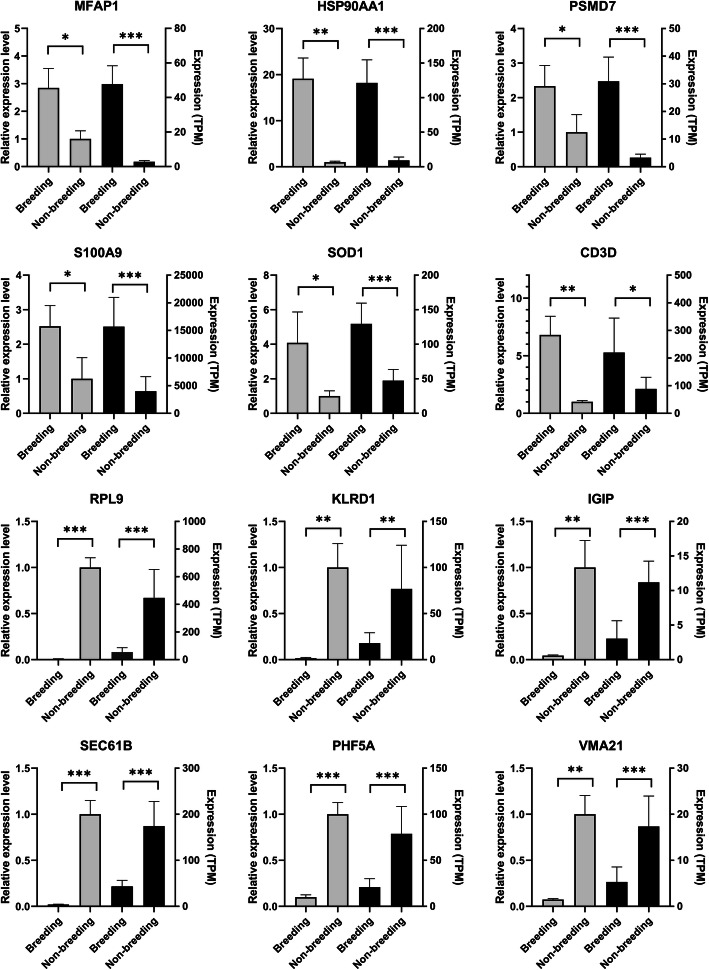
The expression level of genes verified by qRT-PCR. “*” represents the *P*-value< 0.05, “**” represents the *P*-value< 0.01, “***” represents the *P*-value< 0.001. Data were shown as mean ± SD. The left axis represents gene expression levels verified by qRT-PCR. The right axis represents the expression levels in TPM units of RNA-seq

Twelve DEGs (*MFAP1, HSP90AA1, PSMD7, S100A9, SOD1, CD3D, RPL9, KLRD1, IGIP, SEC61B, PHF5A, VMA21*) were selected for verification. As shown in Fig. 10, the results of qRT-PCR indicated similar expression tendencies with transcriptome sequencing. The qRT-PCR validation further improves the reliability of the present study.

## Discussion

Animals in nature need to balance resource allocation between reproduction and self-maintenance, and immunity is a major component of self-maintenance [22]. The reproduction and conservation of giant pandas has been, and continues to be, of global concerns [12, 23]. Our research group’s previous work investigated immune changes at four key phases of female giant panda reproduction [11]. However, the immune performance of male giant pandas during reproduction has been little studied. Here we investigated the immune changes in 8 male giant pandas over the breeding season compared with 5 males in the non-breeding season. We monitored the expression of immune-related genes based on peripheral blood transcriptome. We identified 45 immune-related genes with altered expression, mostly up-regulated, in breeding males compared to non-breeding males.

The GO term enrichment of “translation”, “peptide biosynthetic process” and “structural constituent of ribosome” and KEGG pathway enrichment of “ribosome” were observed in up-regulated genes. DIA revealed that “Genetic Information Processing” was the most impacted pathway and was overall, strongly activated. These results suggest an increased requirement for protein synthesis in breeding male giant pandas. The amplification of protein synthesis was also reported in male freshwater spotted snakehead (*Channa punctatus*) during reproductive phases [24]. The enrichment of the ribosome pathway is consistent with findings in sheep testes, and indicates that the normal function of the ribosome plays an essential role in spermatogenesis [25]. The dramatically up-regulated genes were enriched in spliceosome, which removes noncoding introns from transcribed mRNA precursors, suggesting spliceosome is very important in producing necessary gene products related to male sexual development [26]. Oxidative phosphorylation was another enriched pathway in our study. This pathway is an important ATP-related metabolic pathway and provides energy for male reproduction [26]. For the ‘Sensory System’ subcategory, we uncovered a clear upregulation of “Olfactory transduction” and “Phototransduction”. The male giant pandas may use olfactory and visual cues to assess their sexual partner during the breeding season [27]. The most impacted pathway from DIA analysis was “Fatty acid elongation in mitochondria”. Fatty acid elongation is associated with fatty acid synthesis [28]. The sperm polyunsaturated fatty acid content increases during the breeding season and sperm characteristics are affected by fatty acid composition [29, 30]. The need of fatty acid for spermatogenesis might in part explain the upregulation of fatty acid elongation in breeding male giant pandas. During spermatogenesis, Sertoli cells and germ cells can respond to Notch signaling that is crucial for germ cell differentiation and germ stem cell pool maintenance and migration [31, 32]. During mouse folliculogenesis, Notch signaling was reported to plays an important role in follicular development and angiogenic growth [33]. Moreover, two hub genes *HSPA4* and *SOD1* were 3.36 and 3.25-folder higher in breeding males than non-breeding males, respectively. The expression of *HSPA4* is higher in germ cells of prenatal gonads [34] and SOD1 activity is higher in stallion during the breeding season [35]. This suggests HSPA4 and SOD1 are involved in spermatogenesis and antioxidant protection of sperm in male giant pandas. The up-regulated genes and enriched pathways may indicate that male giant panda reproductive systems prepare for breeding by triggering protein synthesis, energy generating and spermatogenesis.

### Innate immune changes

The innate immune subsystem typically includes pattern recognition receptors, autophagy, antimicrobial peptides, and many cell types (e.g. dendritic cells, macrophages and natural killer cells), establishing the first line of defense against a wide range of invading pathogens [36, 37]. Moreover, the innate immune subsystem is responsible for the activation of the adaptive immune subsystem [36]. During the breeding season, captive male tree lizards reduced innate immunity [3], while the innate immunity showed no change in male Eurasian tree sparrows and temperate bats [6, 38]. Yet, Arabian and Thoroughbred horses presented an increased innate immunity [17]. Here, we explored the alteration of innate immunity from several aspects and found an enhanced innate immunity in male giant pandas.

We found several key genes referred to as pattern recognition receptors (PRRs) were upregulated, including *CLEC4E* (also known as Mincle), *SUGT1* (SGT1 homolog), *HSP90AA1*, *IL1B,* and *GABARAPL1* (LC3 paralog). Pattern recognition receptors mainly include Toll-like receptors (TLRs), C-type lectin receptors (CLRs), and NOD-like receptors (NLRs) [39]. CLRs were found to recognize microorganisms such as viruses, bacteria, and fungi, and then regulate the production of proinflammatory cytokines [39]. *CLEC4E* encodes macrophage-inducible C-type lectin (Mincle) who is a member of the CLRs family [40]. Mincle has been known to recognize dead cells and bacteria [40]. Evidence showed that Mincle was strongly up-regulated after skin injury and irritation, and mediated a severe inflammatory response and the production of IL1B [41]. The up-regulation of Mincle and IL1B in our study may suggest activation of the host immune responses and protection of giant pandas from skin infectious diseases. DIA revealed that NOD-like receptor signaling pathway was activated in male giant pandas during reproduction. NLRs exert function in inflammatory responses and tissue homeostasis [42, 43]. NOD1 as a member of the NLRs family recognizes invasive microbial pathogens which threaten homeostasis by specific peptidoglycans [39]. The SGT1 was reported to positively regulate NOD1 activation and depletion of SGT1 blocks multiple cellular responses caused by NOD1 activation [44]. Besides, HSP90, which is an evolutionarily conserved molecular chaperone, protects NOD1 from degradation and functions as a stabilizer [44, 45]. NLRs can interact indirectly with LC3 through a signaling cascade to regulate autophagy [45, 46]. Autophagy-associated genes (*BECN1, GABARAPL1*) were also up-regulated in our analysis. What’s more, we also found the up-regulation of *NAMPT* (also known as visfatin), which is a cytokine hormone [47]. The *NAMPT* was up-regulated fourfold in adult chicken testis compared with prepubertal chickens, suggesting the critical functions in spermatogenesis and steroidogenesis [47]. In the mouse testis, the expression of visfatin was found to be significantly associated with antioxidant enzymes activities when using dexamethasone treatment [48]. NAMPT inhibition resulted in the decreased production of many proinflammatory mediators in mouse macrophages [49]. Therefore, the up-regulation of SGT1 and NAMPT in breeding male giant pandas may regulate tissue homeostasis, antioxidant enzymes activities, and proinflammatory mediators.

Autophagy is a fundamental intracellular bulk degradation process with multiple roles in innate immune responses and cellular stress [50, 51]. Beclin1 and LC3 encoded by *BECN1*, *GABARAPL1*, respectively, were both up-regulated. Mammalian core autophagy-related proteins mainly involve several functional units, including the PI3K complex that is composed of Beclin1, the LC3 conjugation system and so on [52]. LC3 conjugation system regulates the elongation of the phagophore and promotes the completion of autophagosome formation [46]. During the breeding stage of testicular recovery, the expression of BECN1 and LC3 began to increase in South American plains vizcacha [53]. Tabecka-Lonczynska et al. observed an increase in beclin1 and LC3 synthesis and confirmed the function of autophagy in adult reproductive male European bison [51]. Up-regulated expressions of BECN1 and LC3 suggests the increased demand for maintaining homeostasis in male giant pandas during the period of reproductive activity.

Peroxisomes are crucial metabolic organelles which play central roles in lipid metabolism and ROS turnover [54]. Accumulating evidence suggests a new function for peroxisomes in microbial infection resolution and antiviral response [54, 55]. What’s more, peroxisomes have been pointed to play an important role in cell type-specific metabolic function in the testis and spermiogenesis [56]. Two antioxidant genes *SOD1* and *PRDX5* were up-regulated in the present study. ROS which has recently emerged as a signal factor in innate immune responses, is influenced by the disruption of redox balance in enzymes and subcellular compartments [55, 57]. Kwang et al. demonstrated that SOD1 tightly regulated the generation of ROS during virus infection [57]. Excess ROS production can induce lipid peroxidation, disrupting membrane characteristics of sperm [58]. Elevated expression of the PRDX5 and SOD1 improves the quality of porcine oocytes by modulating the ROS level [59]. Bernard et al. reported that human PRDX5 interacted with, or bound to, PRRs to activate a proinflammatory response [60]. PRDX5 can trigger the expression and release of IL1B [60]. The expression of PRDX5 and IL1B were both up-regulated in giant pandas, confirming an association between PRDX5 and IL1B. Collectively, peroxisomes are essential for the activation of the innate immune system and the normal function of testis in giant pandas.

The proteasome is responsible for poly-ubiquitinated substrates recognition and intracellular proteins degradation [61]. The proteasome system and autophagy are closely interconnected [62]. The proteasome is a multi-subunit protein complex, consisting of a 20S core particle and 19S regulatory particles [62]. Standard 20S proteasomes can be replaced by immunoproteasomes which are activated by PA28 complex in conditions of infection, inflammation, and an intensified immune response [62]. *PSME1* encoding PA28 alpha, one of PA28 complex, was up-regulated about 2.8 fold in giant pandas during the breeding season. Furthermore, the proteasomes generate spliced peptides from major histocompatibility complex type I (MHC class I) molecules and PA28 enhances the presentation of several viral epitopes [63]. Proteasome subunits were reported to increase the immune tolerance of the rhesus monkey during early pregnancy [64]. The upregulation of PA28 may indicate the enhancement of immunity in giant pandas.

NK cells comprise 5–10% of lymphocytes in peripheral blood and vary with age [65]. Natural killer (NK) cells play an immensely significant role in innate immunity by defending against virus infections [36]. CD94, encoded by *KLRD1*, was down-regulated. CD94-NKG2A receptor complex which recognizes MHC class I, is an inhibitor of the cytotoxic activity of NK cells [66]. CD94/NKG2A inhibitory receptor was reported to down-regulate invariant natural killer T cells responses in mice [67]. *PLCG2* (PLC-gamma2) which encodes phospholipase C-gamma2 belongs to PLC-gamma proteins family and was up-regulated. PLC-gamma proteins, serving as cytoplasmic enzymes, are involved in NK cell activation [68]. NK cell cytotoxicity was completely abrogated in PLC-gamma2-deficient cells by 4-h ^51^Cr-release assay [69]. Integration of down-regulated inhibitor and up-regulated activators may imply the partial activation states of NK cells [65]. Shigeru et al. observed the increase of NK cells in pregnant mouse uterus and identified the roles of NK cells in the maintenance of pregnancy [70]. In the testis of macaque and rat, NK cells have an ability for the maintenance of immune privilege and the surveillance for pathological antigens [71]. The increased expression level of NK cells was also reported in breeding horses, suggesting an increased innate immunity of giant pandas during the breeding season [17].

Considered together, these results suggest an enhanced innate immunity in male giant pandas during the breeding season, which is consistent with previous finding [17]. The energy investment in reproduction does not lead to a corresponding decrease innate immune investment. One possible explanation is that the captive pandas are not in a resource-limited environment [3]. Another possible explanation is that the enhancement of innate immunity and cellular immunity can compensate for the decline in the humoral immunity [72, 73].

### Adaptive immune changes

The two typical cellular subsets T and B cells comprise the adaptive immune system [74]. In terms of cellular immunity, male ruffs showed a decreased immunity while tree frogs showed an increased immunity during the breeding season via phytohaemagglutinin challenge test [8, 75]. When it comes to humoral immunity, many studies have documented the changes in the immune system. Male bank voles and Eurasian tree sparrows had lower humoral immunocompetence [7, 38], While the immunoglobulin concentration of the Great Tit increased during breeding in accordance with previous studies on birds [16]. In this study, we also observed some changes in cellular immunity and humoral immunity of male giant pandas.

Several key genes involved in antigen presentation and processing were up-regulated. The DIA results also confirmed this finding. The antigen processing pathway is required for proteasomes which produce peptide fragments of MHC class I ligands [63]. The activation of the proteasome relies on PA28 who enhances the liberation of immunopeptidome [63]. Not only was PA28 up-regulated, but also HSP70 and HSP90 were also up-regulated in our study. HSP70 stimulates antigen cross-presentation of dendritic cells and the immune response of activated NK cells [76]. HSP90 contributes to the translocation of extracellular antigen and associates with peptides implicated as precursors of MHC class I ligands [77]. Our data indicates that male giant pandas may have better antigen presentation and processing compared to non-breeding males.

T cell receptors consist of an antigen-binding subunit (TCRαβ) and three dimers of protein CD3 signaling subunit assemble in a coordinated way [78]. CD3D and CD3G coding genes showed elevated transcript levels in the current study, which are involved in TCR activation [79]. Moreover, Aykut et al. also documented that male horses had higher CD3 expression level during the breeding season [17]. The upregulation of IL1R2 was found in T-cell activation [79], and we found that IL1R2 was up-regulated in the male giant panda. However, we found the down-regulation of IL15. IL15 is an important cytokine in lymphocyte survival [79] as well as T cell proliferation and differentiation [73]. Moreover, some co-receptors are indispensable for the activation of T cells [79]. Therefore, T cells did not show increased proliferative and differentiation in male giant pandas during the breeding season.

B cells can differentiate into plasma cells and secrete immunoglobulins against the pathogen [73]. For male temperate bats, reproduction did not influence the concentration of immunoglobulin G (IgG) [6]. However, for male Eurasian tree sparrows, birds during the breeding stage had lower IgA levels [38]. In our study, we found the down-regulation of *IGIP.* IGIP has the capability of inducing IgA production by B cells [80]. IGIP is primarily produced by dendritic cells and acts as a switch or differentiation factor to regulate IgA [80, 81]. In our study, we found the down-regulation of *IGIP*. The down-regulation of IGIP may indicate the low concentration of IgA and a reduced humoral immunity in male giant pandas during reproduction.

## Conclusions

The present study is the first RNA-seq report on immune profiles of male giant pandas during the breeding season. We identified 45 immune-related genes with altered expression, mostly up-regulated. These genes were related to pattern recognition receptors, autophagy, peroxisome, proteasome, natural killer cell, antigen processing and presentation. Our results suggest an enhanced innate immunity and cellular immunity in male giant pandas during the breeding season, while low humoral immunity was also found. This study provides a foundation for further studies on reproductive immunity in male giant pandas.

## Methods

### Samples

Peripheral blood samples were collected from 13 captive adult male giant pandas housed at the China Conservation and Research Center for Giant Panda, Sichuan Province, China. The eight male giant pandas peripheral blood samples were collected in April when they were in the breeding season. The five male samples were collected in August when they were in the non-breeding season. A routine physical examination of giant pandas was conducted together with blood sampling. Thirteen pandas with ages ranging from 9 to 11 years old were divided into two groups depending on whether or not they were in the breeding season (Table 1).

**Table 1:** Information of samples, including identification name, age and group

Sample	Age	Group
B1	9	Breeding
B2	11	Breeding
B3	9	Breeding
B4	10	Breeding
B5	11	Breeding
B6	11	Breeding
B7	10	Breeding
B8	10	Breeding
N1	11	Non-breeding
N2	11	Non-breeding
N3	11	Non-breeding
N4	13	Non-breeding
N5	14	Non-breeding

No giant pandas were harmed as a result of this research and continued their normal captive existence after completion of the sampling. Before initiation of this project, the China Conservation and Research Center for Giant Panda had consented to provide blood samples and assisted us in this research.

### Library preparation and sequencing

Total RNA from fresh blood was prepared by TRIzol reagent (Invitrogen) and RNeasy kit (Qiagen). We used Nanodrop 8000 Spectrophotometer (Thermo scientific) to evaluate the purity and concentration of RNA. RNA integrity was then checked by using an RNA PicoChip with Agilent 2100 Bioanalyzer (Agilent Technologies). The extracted RNA samples were used for the cDNA synthesis. Double-stranded cDNA was amplified. Sequencing libraries construction, quality control and quantification were performed as per the manufacturer’s instructions using kits from the Illumina Company. The cDNA library was sequenced on the Illumina sequencing platform (HiSeq 2000) using standard procedures. Image analysis and base-calling were performed by the Genome Analyzer Pipeline version 2.0 with default parameters. The 150-bp paired-end reads were generated.

### Quantification and mapping

To perform quality control on the data, all raw reads were processed with an adapter trimming and reads filtering by NGS QC Toolkit version 2.3.3 [82]. Quality reports were generated to ensure that clean data were subjected to further analysis by using FastQC version 0.11.5 (https://www.bioinformatics.babraham.ac.uk/projects/fastqc/). A genome index was built and reads data were mapped on the giant panda reference genome by using HISAT2 version 2.1.0 [83]. The reference genome (v90) and reference annotation were downloaded from the Ensembl website (ftp://ftp.ensembl.org/pub/release-90/).

### Calculating differentially expressed genes (DEGs)

SAM files were generated from alignment program HISAT2 and sorted by SAMtools version 1.7 [84]. SAM files stored mapping information. Sample reads counts file was obtained from BAM files by featureCounts version v1.6.2 [85] which counted the features faster. The expression value of transcripts per million (TPM) was calculated from reads counts. We performed principal components analysis (PCA) on the TPM data matrix of the number of reads by R function prcomp. The PCA plot that showed the clustering information was drawn by R package ggbiplot (https://github.com/vqv/ggbiplot). We then used the counts file as the input to R package edgeR [86]. edgeR performed a method based on the poisson model to infer genes with significant expression differences. The expression fold change (FC) and false discovery rate (FDR) were computed for looking for differentially expressed genes between the two conditions. We set a cut-off of 0.05 for FDR to filter genes for immune-related genes analysis. A cut-off of 1.5 and 0.05 were respectively set for absolute value of log_2_FC and FDR. The filtered genes were DEGs for further analysis.

### Analysis of gene enrichment

To deeply understand the function of these gene sets, genes were annotated and enriched by using the Gene Ontology (GO) and Kyoto Encyclopedia of Genes and Genomes (KEGG) (http://www.genome.jp/kegg) databases. The GO system includes three categories, molecular function, biological process, and cellular component. A web server g:Profiler [87] was used to cluster genes to GO terms and computed the adjusted *P*-value (p.adj) by g:SCS algorithm. The KEGG pathway map analysis, presenting biological interpretation of higher-level systemic functions, was performed by KOBAS 2.0 [88]. A threshold of 0.05 was set for p.adj.

To understand the impact and direction of the pathways, KEGG enrichment analysis was also performed by the Dynamic Impact Approach [89]. The estimate of the dynamic change was represented by the “Impact”. The overall direction of the dynamic change was represented by the “Flux”. Detailed methodology has been reported previously [89]. The data set included Entrez Gene ID, FDR, expression ratio, and *P*-value. We uploaded the data set and chose Human as annotation species. An FDR < 0.05 and a P-value < 0.05 between the breeding and non-breeding were used as cut-off.

### Analysis of protein-protein interaction network

STRING [90] is a database of known and predicted protein-protein interactions. We input immune-related genes into STRING to obtain protein-protein interaction networks. The output of protein-protein interactions was simple texts in tabular form. Cytoscape [91] was used to visualize molecular interaction. We used cytoHubba [92] which is the plugin of Cytoscape to calculate hub genes. The genes were ranked by radiality algorithm. Three additional topological algorithms of betweeness, closeness, and degree were used for verification [93].

### Real-time quantitative PCR (qRT-PCR)

We performed the qRT-PCR to confirm the expression changes of genes. A dozen of top-ranked up-regulated and down-regulated genes were selected. The reference gene was GAPDH, which was same as previous studies [73]. We had six samples (B2, B5, B6, N1, N2, N3) because several samples were lost. The sequences of primers were predicted using Primer-BLAST [94] and listed in Table 2. Primers were synthesized at TSINGKE. Each qPCR reaction system was 10 μL containing 5 μL of 2x M5 Hiper SYBR Premix EsTaq (mei5, Beijing, China), 0.2 μL cDNA, 0.2 μL of forward and reverse primer and nuclease-free water. All reactions were performed on Bio-Rad CFX96 Touch in triplicates. The following program was used: 95 °C for 30 s, followed by 40 cycles of 95 °C/10 s, 60 °C/15 s; then 72 °C for 10 s. The optimized comparative Ct (2^-ΔΔCt^) value method was applied to calculate gene expression levels. We used GraphPad Prism 8.0.2 software to analyze data.

**Table 2:** The primer information of qRT-PCR

Gene	Primer (5′ → 3′)
MFAP1-F	AACCGCCCATTCAGTCTACG
MFAP1-R	ATGCCGGGCCAATCTTTCTT
HSP90AA1-F	TGCGAGGAGCTAATCCCTGA
HSP90AA1-R	TCGTGAATTCCCAGCTTGATGT
PSMD7-F	TGTTGTTGGTGTGCTTTTGGG
PSMD7-R	CATCTTCATCAAAAGGGACTGCAAA
S100A9-F	CCAGCTCAGGGTTACCACAC
S100A9-R	CCATCTTCCTGCGTTCCAAGT
SOD1-F	AGAAGGAAGGTGGGCCTGTT
SOD1-R	TGCACTGGTACAGCCTTGTGT
CD3D -F	AGGCTCTCCAAGGCTGCAAA
CD3D -R	TCTTCCGAGGCCAGTTTTCAC
RPL9-F	TGCTGCGTCTACTGCGAGAATG
RPL9-R	ATGTGATTGAAGTCCCTTCGCA
KLRD1-F	ATCACAGAACTCCAGAAAGGCTC
KLRD1-R	GACAGTGGAGCCATCTTCCC
IGIP -F	GTGTGAACGTTTGTGGCTGG
IGIP -R	CCCCCAAATTGGTCCACAGT
SEC61B -F	GGCACTAATGTGGGCTCGTC
SEC61B -R	TACTGGAACAGGGCCAACCTTGA
PHF5A -F	GCCATCGGAAGACTGTGTGAAA
PHF5A -R	CGCCCCTGGTAAGAGCCATA
VMA21-F	AGCCGCCCGACTTCAGAAATGA
VMA21-R	CCACCGCAACAATAGCAGCA
GAPDH -F	GGTATGACAACGAATTTGGCTACA
GAPDH -R	GATGGAAACAGTGGAGGTCAGGA

## Supplementary Information

**Additional file 1: Table S1.** Differentially expressed genes between the breeding season and the non-breeding season. 1128 genes changed in expression level were detected with a given FDR threshold. The table contains three columns, including GENEID, log_2_FC and FDR.

**Additional file 2: Table S2.** GO enrichment of differentially expressed genes. Up-regulated DEGs were enriched in 69 GO terms and down-regulated DEGs were enriched in 18 GO terms.

**Additional file 3: Table S3.** The score of 36 genes calculated by topological analysis methods. The table contains five columns, including Gene Symbol, Radiality, Betweenness, Closeness, and Degree.

## Data Availability

Raw sequence data are accessible at NCBI under the BioProject accession number PRJNA631846 (https://www.ncbi.nlm.nih.gov/sra/PRJNA631846). The giant panda reference genome (v90) (http://ftp.ensembl.org/pub/release-90/fasta/ailuropoda_melanoleuca/) and reference annotation (http://ftp.ensembl.org/pub/release-90/gtf/ailuropoda_melanoleuca/) were downloaded from the Ensembl website. The accession numbers in Additional file 1 Table S1 and Additional file 2 Table S2 correspond to Ensembl website (https://asia.ensembl.org/index.html).

## Abbreviations

DEGs: Differentially expressed genes
TPM: Transcripts per million
PCA: Principal components analysis
FC: Fold change
FDR: False discovery rate
GO: Gene Ontology
KEGG: Kyoto Encyclopedia of Genes and Genomes
PRRs: Pattern recognition receptors
TLRs: Toll-like receptors
CLRs: C-type lectin receptors
NLRs: NOD-like receptors
MHC class I: Major histocompatibility complex type I
NK: Natural killer
IgG: Immunoglobulin G
